# Hyper-maturity and accelerated aging in the hippocampus of mouse models of neuropsychiatric disorders with anxiety-like behavior

**DOI:** 10.1038/s41386-025-02237-6

**Published:** 2025-10-27

**Authors:** Hideo Hagihara, Hisatsugu Koshimizu, Satoko Hattori, Hirotaka Shoji, Miho Tanaka, Kazutaka Ikeda, Tsuyoshi Miyakawa

**Affiliations:** 1 Division of Systems Medical Science, Center for Medical Science, Fujita Health University, Toyoake, Aichi Japan; 2 Office of Research Administration, Fujita Health University, Toyoake, Aichi Japan; 3 Research Creation Support Center, Aichi Medical University, Nagakute, Aichi Japan; 4 Addictive Substance Project, Tokyo Metropolitan Institute of Medical Science, Setagaya-ku, Tokyo Japan; 5 Department of Neuropsychopharmacology, National Institute of Mental Health, National Center of Neurology and Psychiatry, Kodaira, Tokyo Japan

**Keywords:** Anxiety, Gene expression analysis, Senescence

## Abstract

Proper maturation of neuronal and glial cells in the hippocampus is essential for emotional regulation and cognitive function. While pseudo-immaturity, defined as arrested or reversed development, has been extensively implicated in various neuropsychiatric conditions, the opposite phenomenon, hyper-maturity, remains underexplored. Here, we present transcriptomic evidence of hippocampal hyper-maturity across 17 datasets from 16 mouse models with genetic, pharmacological, or other experimental manipulations, identified through a comprehensive screening of over 260,000 omics datasets. These models were characterized by a pronounced overrepresentation of gene expression changes typically observed during postnatal development and included serotonin transporter knockout mice, glucocorticoid receptor overexpressing mice, and corticosterone-treated mice, models of depression and anxiety, Df(16)A^+/−^ mice, a 22q11.2 deletion schizophrenia model, β-glucuronidase-deficient lysosomal storage disorder model mice, and senescence-prone SAMP8 mice. Meta-analysis of enriched pathways highlighted associations of synapse-related genes with the hyper-maturity signature. Behavioral annotations from public datasets further suggest that hippocampal hyper-maturity models predominantly exhibit increased anxiety-like behaviors, whereas immaturity models tend to display the opposite pattern. Notably, hippocampal hyper-maturity encompassed two transcriptional dimensions: enhanced postnatal development and accelerated aging. For example, SAMP8 mice aligned more with developmental enhancement, whereas corticosterone-treated and lysosomal storage disorder models reflected aging acceleration. Combined analysis with available single-cell RNA-sequencing data further delineated that microglia and granule cells may contribute to aging-associated transcriptional shifts. These findings suggest that hippocampal hyper-maturity and accelerated aging represent convergent molecular phenotypes associated with anxiety-like behavior. Bidirectional alterations in hippocampal maturity may serve as a transdiagnostic endophenotype and offer novel therapeutic or anti-aging targets for neuropsychiatric disorders.

## Introduction

Accumulating evidence suggests that the maturation states of brain cells can deviate dynamically and plastically from typical trajectories due to various genetic and environmental factors. Previously, we identified the immature dentate gyrus (iDG) phenotype in the hippocampus of calcium/calmodulin-dependent protein kinase II alpha knockout (Camk2a KO) mice [1]. In this phenotype, the majority of principal neurons in the hippocampal DG are arrested in a pseudo-immature state during adulthood, exhibiting molecular, morphological, and physiological characteristics that closely resemble those of typically developing infant wild-type mice. As molecular characteristics, mature neurons typically exhibit reduced expression of mature neuronal markers and/or increased expression of immature neuronal and cell cycle markers [2]. To date, this pseudo-immaturity phenotype in the DG has been observed in subsets of patients with schizophrenia (SZ), bipolar disorder (BD) [3], Alzheimer’s disease (AD) [4], and epilepsy [5, 6], as well as in several mouse strains that display behavioral abnormalities associated with neuropsychiatric disorders [7–13]. Importantly, matured DG neurons in adult mice can revert to a pseudo-immature state under various conditions, such as antidepressant treatment and neuronal hyperexcitation—a phenomenon referred to as “dematuration” or “rejuvenation” [2, 14–18]. Furthermore, evidence from our group and others has shown that pseudo-immaturity can also be observed in other cell types and brain regions, such as the prefrontal cortex and amygdala, in both mice and human patients with neuropsychiatric disorders [19–23]. These findings suggest that maturational states of certain brain cells can change plastically in response to various genetic and environmental insults. However, knowledge of the opposite phenomenon, or hyper-maturation, remains limited. A few studies have reported hyper-maturation in specific cell types or brain regions, such as parvalbumin-expressing interneurons in the visual cortex of methyl-CpG-binding protein 2-deficient mice, a model for the neurodevelopmental disorder Rett syndrome [24], and the cingulum bundle in patients with autism spectrum disorder [25]. This gap in knowledge highlights the need for more comprehensive studies to determine whether hyper-maturation is restricted to specific conditions or represents a broader biological process induced widely by diverse factors.

In this study, we present transcriptomic evidence supporting the presence of hyper-maturity and accelerated aging in the hippocampus across various mouse models subjected to genetic, pharmacological, and other experimental manipulations. This was accomplished through a comprehensive analysis of BaseSpace, an omics database containing over 260,000 publicly available datasets [26]. Meta-analyses of these models further suggest that synaptic alterations and increased anxiety-like behavior may be associated with the hippocampal hyper-maturity phenotype. Furthermore, integration of available single-cell RNA-sequencing data from the aging mouse DG revealed cell-type-specific contributions to transcriptomic signatures of accelerated aging. Together, these findings shed new light on the possibility that plastic and bidirectional changes in hippocampal maturation, including not only pseudo-immaturity but also hyper-maturity, may underlie the neural basis of anxiety-related neuropsychiatric disorders.

## Materials and methods

### Animals

For RNA-sequencing analysis, we used adult male mice (16–17 weeks old) from three genotypes: serotonin transporter (Sert) homozygous KO, heterozygous KO, and wild-type littermates (*n* = 5 per genotype) [27, 28]. Additionally, male C57BL/6J mice (16 weeks old) were treated with either corticosterone or vehicle for 40 days (*n* = 5 per group) [29]. All animal experiments were approved by the Institutional Animal Care and Use Committee of Fujita Health University and the Tokyo Metropolitan Institute of Medical Science.

### RNA sequencing

The DG was dissected as previously described [30]. For corticosterone-treated mice, the dissected DG was bisected near the midline and further divided into the dorsal and ventral parts. Total RNA was extracted using the RNeasy plus micro kit (Qiagen, Tokyo, Japan), and 10–100 ng of total RNA was used for library preparation. RNA-seq libraries were constructed using the NEBNext Ultra RNA Library Prep Kit for Illumina, following the manufacturer’s protocols (Illumina, San Diego, CA), and sequencing was performed on an Illumina HiSeq or MiSeq platform. Differentially expressed gene (DEG) lists for Sert homozygous KO mice, Sert heterozygous KO mice, and corticosterone-treated mice are provided in Tables S1–S4. Genes with absolute fold change >1.2 and raw *P* value < 0.05 were imported to the web-based bioinformatics tool BaseSpace (Illumina, San Diego, CA; https://basespace.illumina.com) [26], in accordance with the manufacturer’s instructions.

### Identification of mouse models of hippocampal hyper-maturity

We comprehensively queried gene expression datasets of animal models exhibiting hippocampal hyper-maturity using the BaseSpace database [26]. BaseSpace employs the Running Fisher algorithm, which utilizes a normalized, fold change-based ranking approach. This method allows for cross-study comparability among datasets derived from different species and analytical platforms (e.g., DNA microarray and RNA sequencing) [31, 32]. For the analysis, we used seven datasets representing developmental gene expression changes in the hippocampal DG of naive wild-type mice at various ages (8, 11, 14, 17, 21, 25, and 29 days old) compared to adults (34–36 weeks old) [17]. The objective was to identify, from over 260,000 omics datasets, hippocampal datasets that show a positive correlation with DG developmental profiles, reflecting progressive cellular maturation toward a hyper-mature state. Query results were filtered using the keyword “Hippocampus” and an overlap P-value threshold of <1.0 × 10^−10^. Data derived from embryos, infant animals, or cultured cells were excluded, as the study focused specifically on adult animal brain tissue.

### Pathway enrichment meta-analysis

**Fig. 1:**
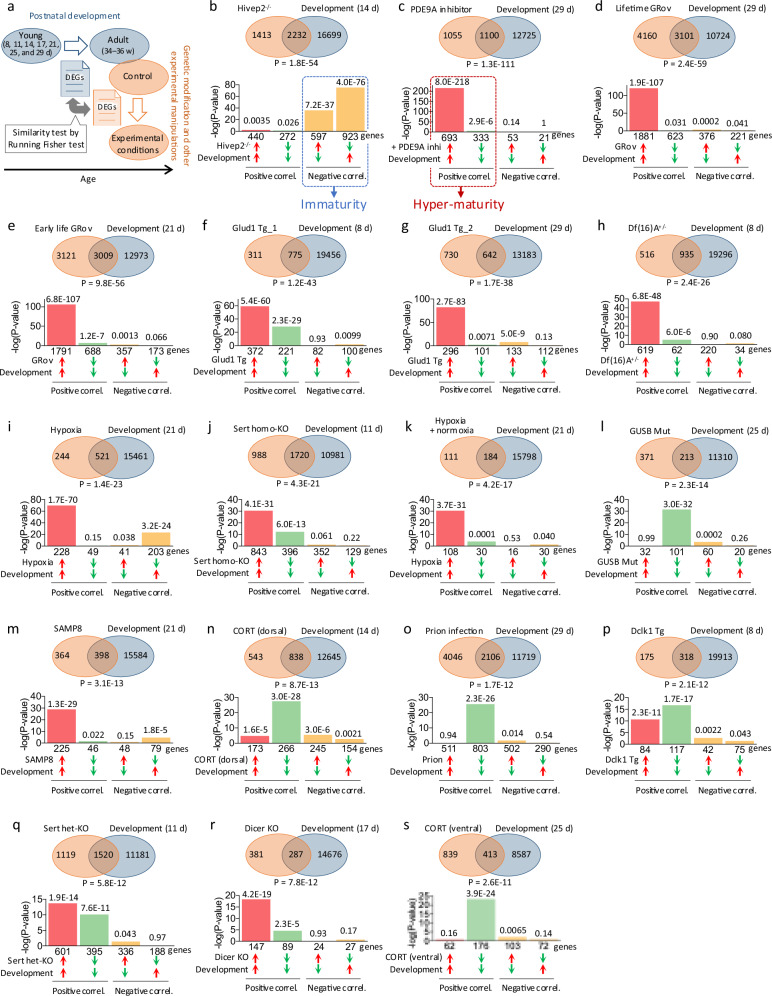
Transcriptomic evidence for hyper-maturity in the mouse hippocampus. **a** Patterns of differentially expressed genes (DEGs) in the hippocampus of particular strains/conditions of animals (mutants/conditions compared to wild-type/control animals) were compared with those in the DG of typically developing wild-type mice (8, 11, 14, 17, 21, 25, and 29 days old (d) young mice) compared to adults (34–36 weeks old (w)); GSE42778 and GSE113727) using Running Fisher test. **b** An example of transcriptomic immaturity. Gene expression in the DG of adult human immunodeficiency virus type I enhancer binding protein 2 (Hivep2) KO mice (DEGs between KO and wild-type mice) was compared with that of 14-day-old mice (DEGs between 14-day-old infant and 34–36-week-old adult mice). **c**–**s** Transcriptomic hyper-maturation observed in 17 hippocampal datasets from 16 mouse strains/conditions. Gene expression pattern in the hippocampus of PDE4A inhibitor-treated mice (**c**), the DG of GRov throughout the lifetime (**d**) and in early life (**e**) the hippocampus of Glud1 transgenic (Tg) mice (**f**, **g**), the hippocampus of Df(16)A^+/−^ mice (**h**), the hippocampus of mice exposed to hypoxic conditions (**i**) and then normoxia (**k**), the DG of Sert homozygous (**j**) and heterozygous (**q**) KO mice, the hippocampus of GUSB mutant mice (**l**) the hippocampus of SAMP8 mice (**m**), the dorsal (**n**) and ventral (**s**) DG of corticosterone (CORT)-treated mice, the hippocampus of ME7 prion-infected mice (**o**), the hippocampus of Dclk1 Tg mice (**p**), and the hippocampus of Dicer KO mice (**r**) were compared with that in the DG of young mice of indicated age. The *P* values shown below each Venn diagram indicate the overlap *P* values for the comparison between the two datasets. The bar graphs below show the breakdown of the common genes between the two datasets, and the numbers above each bar represent the overlap *P* values for each comparison. These identified model mice are listed in ascending order of their overlap *P* values with the developmental data.

Pathways and biological groups enriched among the genes of interest were identified using BaseSpace, which integrates rank-based enrichment statistics with biomedical ontologies. We conducted a meta-analysis of gene sets representing hyper-maturity or accelerated aging (i.e., gene sets showing positive correlations, denoted by red dashed square in Figs. 1c and 3c, respectively), to discover which biomedical ontologies are significantly regulated in common across these gene sets. Each gene set of interest was manually extracted from the full gene lists and uploaded to BaseSpace. Using the BaseSpace platform, we selected the gene sets of interest and performed meta-analysis for pathway enrichment. The analysis included biological processes from Gene Ontology and canonical pathways curated in the Broad MSigDB database.

### Maturity index and anxiety index

#### Maturity index

The calculated overlap *P* values were transformed to derive the maturity index using the following formula [13, 21]:

- For positive correlations:
  Maturity index = −log10([overlap *P* value between the DG development dataset and the screened dataset]).
- For negative correlations:
  Maturity index = −log10(-1 × [overlap *P* value between the DG development dataset and the screened dataset]).

#### Anxiety index

Effect sizes (d) for each anxiety-related measure comparing model mice to controls were used as the anxiety index. The d value for each mouse model and each measure was calculated as follows:

$${{{\rm{d}}}}=({{{\rm{M}}}}_{{{\mathrm{models}}}}{{{\rm{\hbox{--}}}}}{{{\rm{M}}}}_{{{\mathrm{controls}}}})/{{{\rm{S}}}}{{\mathrm{pooled}}}$$

$${{{\rm{S}}}}{{\mathrm{pooled}}}={[({{S}^{2}}_{{{\mathrm{model}}}}+{{S}^{2}}_{{{\mathrm{control}}}})/2]}^{1/2},$$

where *M* denotes the mean and *S* denotes the standard deviation (SD).

We collected data on behavioral tests assessing anxiety-related behaviors from as many animal models as possible, as referenced in this study, using published papers and in-house studies (see Table S24). Literature searches were conducted in PubMed and Google Scholar using relevant keywords, including the name of the strain or experimental condition, species (mice or rats), “behavior,” and “anxiety.” From the top search results, we prioritized data presented as actual values of mean and SD or standard error of the mean (SEM). For some behavioral measures, mean and SD or SEM values were estimated from graphs presented in the papers.

### Neural excitation, neural suppression, and corticosterone indices

We used transcriptome data from the hippocampal DG of pilocarpine-induced epileptic rats (GSE47752; Table S5) [33], available in the BaseSpace database, as a representative model of neural excitation. A total of 6777 DEGs were identified between pilocarpine-treated rats (1 day after treatment) and saline-treated controls using thresholds of absolute fold change >1.2 and raw *P* < 0.05; these genes were considered to be associated with neural excitation. As a dataset reflecting neural activity suppression, we used 73 genes specifically upregulated in response to activity suppression by tetrodotoxin, a sodium channel blocker, in primary mouse cortical neurons (GSE90988; Table S5) [34]. This gene set was imported into the BaseSpace platform and used for the subsequent analysis. Data from corticosterone-treated mice were incorporated as described above. The expression patterns of these neural excitation-, neural suppression-, and corticosterone-associated genes were then compared with those of hyper-maturity and immaturity model mice using the BaseSpace platform. Statistical similarity was evaluated with the Running Fisher test. Based on the resulting overlap P-values, we calculated a neural excitation index, a neural suppression index, and a corticosterone index for each mouse model, using formulas analogous to those described above for the maturity index in the “Maturity index and anxiety index” section.

### Transcriptome data of diazepam-treated mice

We used a transcriptome dataset obtained from the hippocampus of mice chronically treated with benzodiazepine diazepam (NCBI GEO accession number GSE76700; Table S5) [35]. In that study, adult wild-type mice received diazepam at a dose of 5 mg/kg twice daily for 10 days, after which hippocampal gene expression was analyzed using microarray. DEGs were identified by comparing diazepam-treated mice with control mice using cutoffs of absolute fold change >1.2 and raw *P* < 0.05. The resulting DEG list was manually uploaded to the BaseSpace platform. This diazepam dataset was then compared with the aforementioned mouse development and aging datasets, as well as with the hyper-maturity model mice, using the BaseSpace platform. Statistical similarity was assessed using the Running Fisher test.

### Cell-type contribution analysis

We used publicly available single-cell RNA-sequencing data to assess cell-type-specific aging-related gene expression changes in the mouse DG [36]. The study identified 11 distinct cell types: astrocyte, quiescent neural stem cell, active neural stem/progenitor cell, neuroblast/immature neuron, granule cell (GC), Cajal–Retzius-like cells, oligodendrocyte precursor cell, endothelial cell, pericyte, smooth muscle cell (SMC), and microglia. For each cell type, we extracted DEGs between old (16–21 months old) and young adult (3 months old) mice using a threshold of absolute fold change >1.2 and raw *P* value < 0.05. These 11 DEG lists were imported to the BaseSpace and compared with expression patterns of signature genes of accelerated aging (the overlapping region shaded with red stripes in the Venn diagram of Fig. 5a). Overlap *P* values were calculated and transformed into the negative log10 scale (−log10 [overlap *P* value]), with higher values indicating a greater contribution of the corresponding cell type to the transcriptomic aging signature in the mouse models.

### Human transcriptome data

#### Disease data

We used transcriptome datasets obtained from the hippocampus of SZ, BD, and major depressive disorder (MDD) from three studies [37–39] (Table S5). For the study by Lanz et al. (NCBI GEO accession number GSE53987) [38], we used the dataset available on the BaseSpace platform. For the studies by Jaffe et al. (SRA accession number SRP241159) [39] and Kohen et al. (NCBI GEO accession number GSE42546) [37], we downloaded the raw data from the respective repositories and manually uploaded DEG lists to BaseSpace. These lists were generated by comparing patients and controls using an absolute fold change >1.2 and raw *P* < 0.05 as cutoffs.

#### Development and aging data

Transcriptome data for human hippocampal development and aging were obtained from NCBI GEO (accession code GSE25219) [40] (Table S5). For the developmental dataset, DEGs were extracted by comparing individuals aged 20–39 years with those aged 0–5 months. For the aging dataset, DEGs were extracted by comparing individuals over 60 years old with those aged 20–39 years. In both cases, gene lists were filtered using a cutoff of absolute fold change >1.2 and raw *P* < 0.05, and were manually uploaded to the BaseSpace platform. These developmental and aging datasets were then compared with the above human disease datasets using the BaseSpace platform, and statistical similarity was evaluated using the Running Fisher test.

### Statistical analysis

Overlap *P* values between the given two datasets were calculated using the Running Fisher algorithm provided on the BaseSpace platform. Linear regression analysis was performed using GraphPad Prism 8 (version 8.4.2; GraphPad Software, San Diego, CA).

## Results

### Transcriptomic evidence of hyper-maturity in the hippocampus

To identify animal models exhibiting hippocampal hyper-maturity, we performed a comprehensive cross-study transcriptomic screening using the BaseSpace database. By querying over 260,000 publicly available omics datasets with a developmental gene expression signature derived from the DG of postnatal wild-type mice, we identified datasets showing strong concordance with progressive cellular maturation. Similarity between DEG patterns was evaluated using the Running Fisher algorithm, which ranks genes based on normalized fold change (Fig. 1a). As expected, a representative iDG model, Hivep2 KO mice [7], exhibited a significant negative correlation with the DG developmental trajectory (Fig. 1b), supporting the presence of pseudo-immaturity in the DG from a transcriptomic perspective. Through this comprehensive screening, we initially identified 13 publicly available datasets that met our criteria and showed positive correlations with at least one of the seven DG development datasets (Fig. 1c–s, excluding 1j, n, q, s), indicating a transcriptomic hyper-maturity phenotype. We further validated this phenotype using four datasets generated in the present study from mouse models exhibiting increased anxiety-like behavior, specifically Sert homozygous and heterozygous KO mice (Fig. 1j, q) and corticosterone-treated mice (dorsal and ventral DG, Fig. 1n, s). Details regarding the relationship between anxiety-like behavior and hippocampal hyper-maturity are provided in the section below titled “Increased anxiety-like behavior in mouse models with hippocampal hyper-maturity”.

In total, 17 datasets from 16 mouse strains or experimental conditions were identified as models of hippocampal hyper-maturity (Fig. 1c–s, Table S5). Among these, mice chronically treated with PF-04447943, a selective phosphodiesterase 9A (PDE9A) inhibitor, exhibited the lowest overlap *P* value (i.e., the highest degree of similarity with developmental data) (GSE36237; Fig. 1c). The overall results of the comparison with each of the seven DG developmental time points are shown in Fig. 4a. Genes/transcripts whose expression changed in the same and opposite directions together in the two datasets were denoted as showing a positive and negative correlation, respectively. Furthermore, mice with hippocampal hyper-maturity included: mouse strains that overexpress the glucocorticoid receptor (GRov) throughout the lifetime (Fig. 1d) and in early life (Fig. 1e) (GSE30187); transgenic mice with neuron-specific (under the control for enolase promoter) overexpression of glutamate dehydrogenase 1 (Glud1 Tg_1, GSE48911; Glud1 Tg_2, GSE11419), independent studies from the same group; Fig. 1f, g; Df(16)A^+/−^ mice, a model of human 22q11 microdeletion syndrome (Fig. 1h; GSE10784); mice exposed to hypoxic conditions (8% O_2_) for 3 h (Fig. 1i) and then reoxygenation for 1 h (Fig. 1k) (GSE19709); Sert homozygous KO (Fig. 1j) and heterozygous KO mice (Fig. 1q); β-glucuronidase (GUSB) mutant mice with a lysosomal storage disorder (mucopolysaccharidosis VII)-related mutation (GSE34071; Fig. 1l); senescence-accelerated prone mice 8 (SAMP8, GSE65877; Fig. 1m); a corticosterone-induced mouse model of depression (dorsal and ventral DG, Fig. 1n, s); mice infected with ME7 prion (GSE23182; Fig. 1o); transgenic mice overexpressing a splice-variant of the doublecortin-like kinase-1 gene (Dclk1, GSE8349; Fig. 1p); and neuron-specific Dicer KO mice (Camk2a-CreERT2/Dicer 1-floxed mice, GSE61937; Fig. 1r). The genes with commonly altered expression in the hippocampus or DG of the above-mentioned hyper-maturity model mice and the DG development datasets are listed in Tables S6–S22.

### Predominant association of hyper-maturity genes with synaptic alterations

We defined genes showing positive correlation as feature genes of hyper-maturity (groups of genes denoted with red dashed square in Fig. 1c). Pathway enrichment meta-analysis of 17 gene sets of hyper-maturity feature genes revealed a predominant enrichment for synapse-related terms, such as “synaptic membrane,” “synaptic signaling,” “asymmetric synapse,” and “postsynaptic specialization” (Table S23a), suggesting that synaptic alterations are the most relevant biological process to hyper-maturity in the hippocampus.

The 17 identified datasets were classified into two groups based on their gene expression patterns. Twelve datasets belonged to the group characterized by a predominant overlap of genes upregulated in both the hyper-maturity models and development (represented by red bars in the bar graph; Fig. 1c–k, m, q, r). The remaining five datasets were classified into the group showing a predominant overlap of genes downregulated in both the models and development (green bars in the bar graph; Fig. 1l, n–p, s). To explore potential differences in biological processes between these two groups, we performed pathway enrichment analysis for each group separately. The results showed that the first group was enriched for synapse-related terms (Table S23b), suggesting excessive synaptic organization. The latter group was enriched for terms related to extracellular matrix, cell adhesion, and blood vessel (Table S23c), indicating alterations in extracellular connectivity and vascular system. These findings suggest that although these models share the hyper-maturity phenotype, they may involve distinct underlying biological processes.

### Increased anxiety-like behavior in mouse models with hippocampal hyper-maturity

**Fig. 2:**
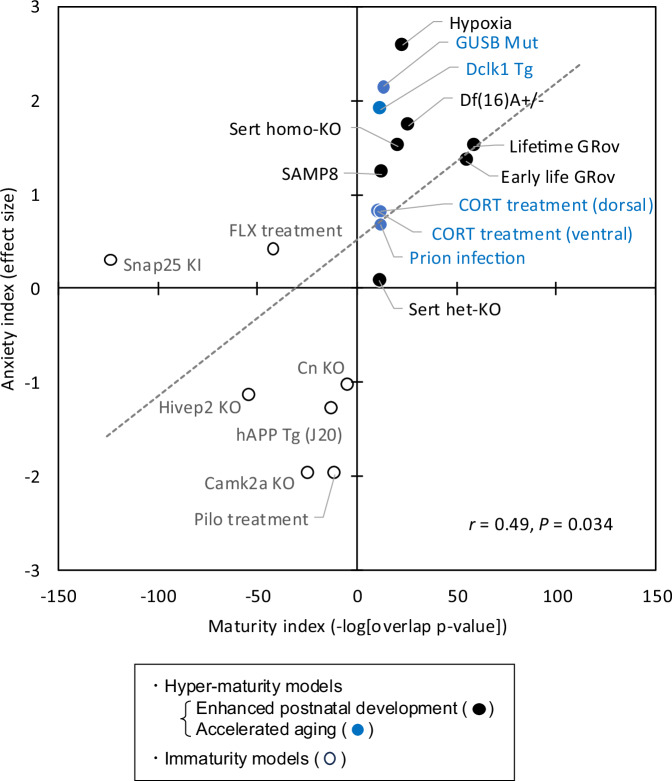
Hippocampal hyper-maturity correlated with increased anxiety-like behavior. Scatter plot showing a positive correlation between the maturity index and the anxiety index. Filled symbols represent hyper-maturity mouse models (black: mice with enhanced postnatal development; blue: mice with accelerated aging), while open symbols indicate immaturity mouse models. The dashed line fitted to all 19 data points. Camk2a KO calcium/calmodulin-dependent protein kinase II alpha knockout mice, Cn KO calcineurin knockout mice, FLX treatment mice treated with fluoxetine, hAPP Tg (J20) human amyloid precursor protein transgenic mice (line J20), Hivep2 KO human immunodeficiency virus type I enhancer binding protein 2 knockout mice, Pilo treatment mice treated with pilocarpine, Snap25 KI dominant-negative synaptosomal-associated protein, 25 kDa knock-in mice.

Given that the hippocampus plays a crucial role in regulating anxiety [41], we investigated how mouse models with hippocampal maturation abnormalities exhibit anxiety-related behaviors. A literature review revealed that mouse models with hippocampal hyper-maturity displayed increased anxiety-like behaviors, as assessed by tests such as the light-dark transition test and the elevated-plus maze test (Table S24). For instance, GRov mice exhibited a longer latency to first enter the light box in the light-dark transition test, and Sert homozygous KO mice spent less time in the open arms in the elevated-plus maze test compared to their respective control mice, both reflecting increased anxiety-like behaviors. In contrast, mouse models with hippocampal immaturity generally exhibited decreased anxiety-like behaviors, with some exceptions (Table S24). For example, Camk2a KO mice, Hivep2 KO mice, and hAPP Tg AD model mice spent more time in the open arms in the elevated-plus maze test, indicating decreased anxiety-like behavior. Another hippocampal immaturity model, Snap25 Tg mice, displayed a shorter latency to first enter the light box (indicative of decreased anxiety), while spending less time in the light box (indicative of increased anxiety), suggesting inconsistent anxiety-related behaviors. To explore the relationship between hippocampal maturation and anxiety, we calculated a maturity index and an anxiety index for each hyper-maturity and immaturity mouse model (see “Materials and methods” for details) and plotted these mouse models in two-dimensional space based on these indices. Linear regression analysis revealed a significant negative correlation between the two indices across mouse models (Pearson’s *r* = 0.49, *p* = 0.034; Fig. 2), suggesting that hippocampal hyper-maturity and immaturity are associated with increased and decreased anxiety-like behavior, respectively.

Given the suggested link between anxiety-related behavior and changes in hippocampal maturity, we next investigated the effects of anxiolytic treatment on hippocampal maturation and its relationship with hyper-maturity models. We utilized a transcriptome dataset obtained from the hippocampus of mice chronically treated with diazepam, a benzodiazepine that potentiates gamma-aminobutyric acid type A receptor (GABA_A_ receptor) activity [35]. This dataset did not show strong correlations with gene expression patterns associated with postnatal development, but it did exhibit a moderate positive correlation with those of 29-month-old aged mice, suggesting an accelerated aging phenotype (Fig. S1a, b; see below for details on comparison with aged mice and accelerated aging). Furthermore, it showed either negative correlations (Fig. S1c–f) or positive correlations (Fig. S1g, h) in gene expression patterns with several hyper-maturity models characterized by increased anxiety-like behaviors. In certain hyper-maturity model mice showing negative correlations, specifically lifetime GRov mice, Glud1 Tg mice, and Sert-homo KO mice, chronic diazepam treatment could be beneficial for normalizing their gene expression abnormalities; however, this hypothesis needs to be experimentally tested by administering diazepam directly to those models.

### Exploring potential mechanisms contributing to hippocampal hyper-maturity

To explore potential mechanisms by which diverse manipulations (e.g., genetic modifications, pharmacological treatments, pathological conditions) converge on the hippocampal hyper-maturity phenotype, we examined several hypotheses. Since neural hyperexcitation is a major factor contributing to hippocampal immaturity [13, 15, 16, 21, 42], we hypothesized that suppression of neural activity might instead underlie hyper-maturity. In addition, because chronic administration of corticosterone, a stress hormone, induced hippocampal hyper-maturity, we also considered a potential link with stress exposure. We calculated a neural excitation index, a neural suppression index, and a corticosterone index for each hyper-maturity model, as well as for immaturity models, using the same method applied to compute the maturity index in Fig. 2. The relationships between these indices and the maturity index are shown in Fig. S2. As also shown in Fig. 2, the maturity index clearly distinguished hyper-maturity models, which exhibited higher values, from immaturity models, which had lower values. The neural excitation, neural suppression, and corticosterone indices varied across hyper-maturity models and did not provide clear separation from immaturity models (Fig. S2). These findings suggest that neither reduced or suppressed neural activity nor corticosterone-related stress exposure is likely to account for the hippocampal hyper-maturity phenotype, although these hypotheses were evaluated with a limited number of datasets.

### Two dimensions of hippocampal hyper-maturity: enhanced postnatal development and accelerated aging

**Fig. 3:**
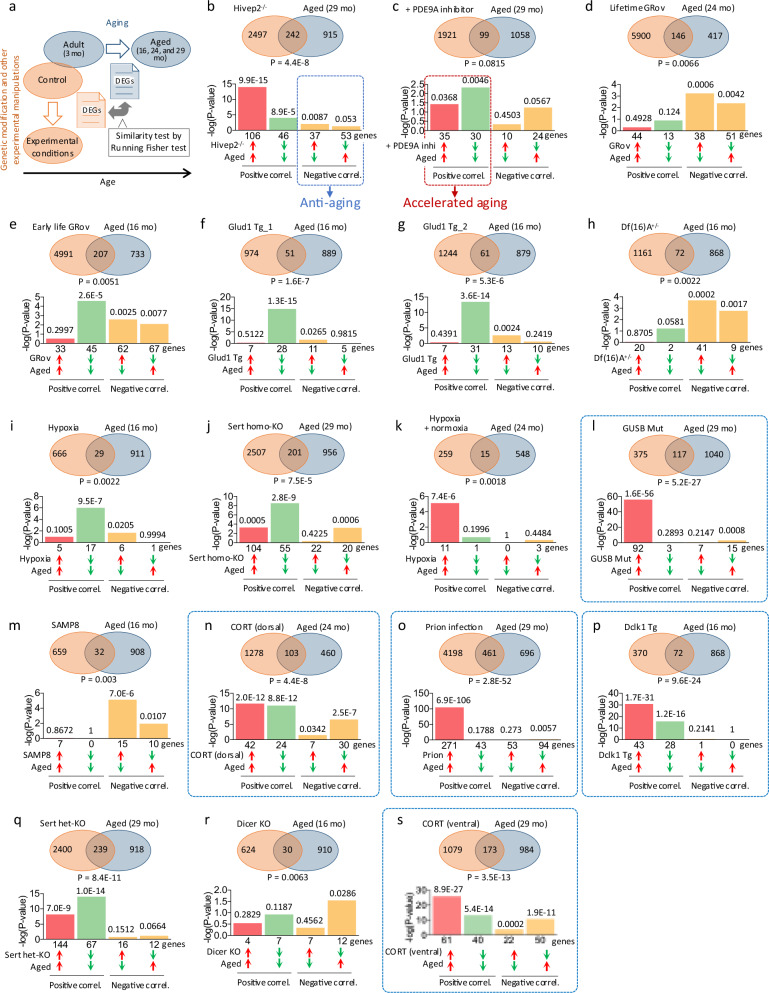
Comparison of gene expression patterns between mouse models of hippocampal hyper-maturity and aged mice. **a** Patterns of differentially expressed genes (DEGs) in the hippocampus of hyper-maturity model mice (mutants/conditions compared to wild-type/control animals) were compared with those in the hippocampus of typically aging wild-type mice (16, 24, and 29 months old (mo) mice) compared to adults (3 mo); GSE20270 and GSE61915) using the Running Fisher test. **b** An example of a comparison between a hippocampal immaturity model (DEGs between Hivep2 KO and wild-type mice) and aged mice (DEGs between 29-month-old aged and 3-month-old adult mice). **c**–**s** Comparisons between hippocampal hyper-maturity models (DEGs between mutants/conditions and wild-type/control mice) and aged mice (DEGs between 16–29-month-old aged and 3-month-old adult mice). The order of the hyper-maturity models is consistent with that shown in Fig. 1.

Since both postnatal development (from infancy to adulthood) and aging (from adulthood to old age) are continuous biological processes associated with the progression of age, we investigated how aging-related gene expression changes are related to hyper-maturity. Gene expression patterns from the identified 17 datasets were compared with those of aged hippocampus samples (16, 24, and 29 vs. 3 months old; GSE20270 and GSE61915) (Fig. 3a). A high degree of similarity (overlap *P* value < 1.0 × 10^−10^) with positive correlation was observed in five hyper-maturity models: GUSB mutant, prion-infected, Dclk1 Tg, Sert heterozygous KO, and corticosterone-treated (ventral DG) mice (Fig. 3). We again observed two groups of datasets based on their gene expression patterns. Six models showed a predominant overlap of genes upregulated in both the hyper-maturity models and aging (red bars in the bar graph; Fig. 3k, l, n–p, s), whereas seven datasets showed a predominant overlap of genes downregulated in both (green bars; Fig. 3c, e–g, i, j, q). Pathway enrichment analysis revealed that the former group was enriched for inflammation-related terms (Table S25a), suggesting increased inflammation, while the latter group showed enrichment for various non-specific biological categories (Table S25b). These findings suggest that the two groups of models may reflect distinct aspects of aging-related biological processes.

**Fig. 4:**
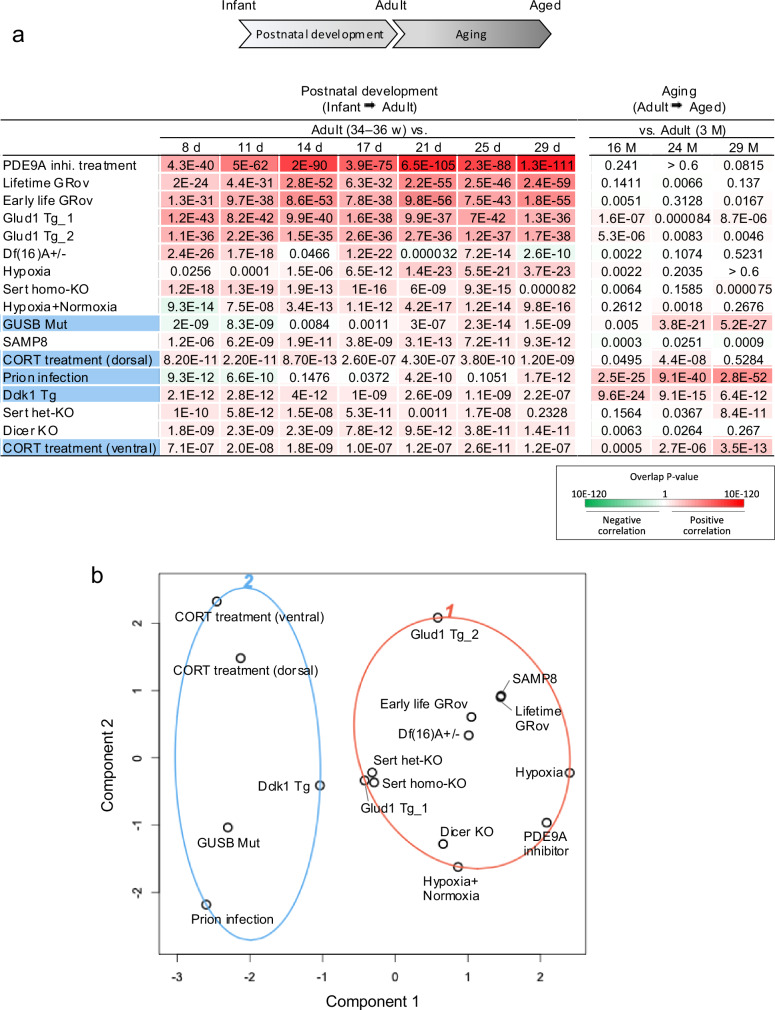
Two dimensions of hippocampal hyper-maturity: enhanced postnatal development and accelerated aging. **a** A matrix of overlap *P* values between each pair of datasets. The gene expression patterns of the identified hyper-maturity model mice were compared with those at each time point of postnatal development and aging using the BaseSpace platform, and overlap *P* values were calculated using the Running Fisher test. **b** PCA scatter plot based on the overlap *P* values. The first two principal components explain 49.8% of the variance. Red and blue circles indicate Group 1 and Group 2, respectively, as identified by PCA.

PCA based on overlap *P* values across datasets classified 17 datasets into two distinct groups: one predominantly associated with enhanced postnatal development (Group 1, 12 models) and the other with accelerated aging (Group 2, five models) (Fig. 4). All five datasets included in PCA Group 2 were those that exhibited relatively high overlap in the genes downregulated in both the hyper-maturity models and during postnatal development (Fig. 1l, n–p, s). These findings suggest that hyper-maturity encompasses two dimensions: overrepresentation of transcriptomic systems related to postnatal development and accelerated aging in adulthood, with distinct mouse models classified into each group.

### Cell-type contributions to accelerated aging-related gene expression signature

**Fig. 5:**
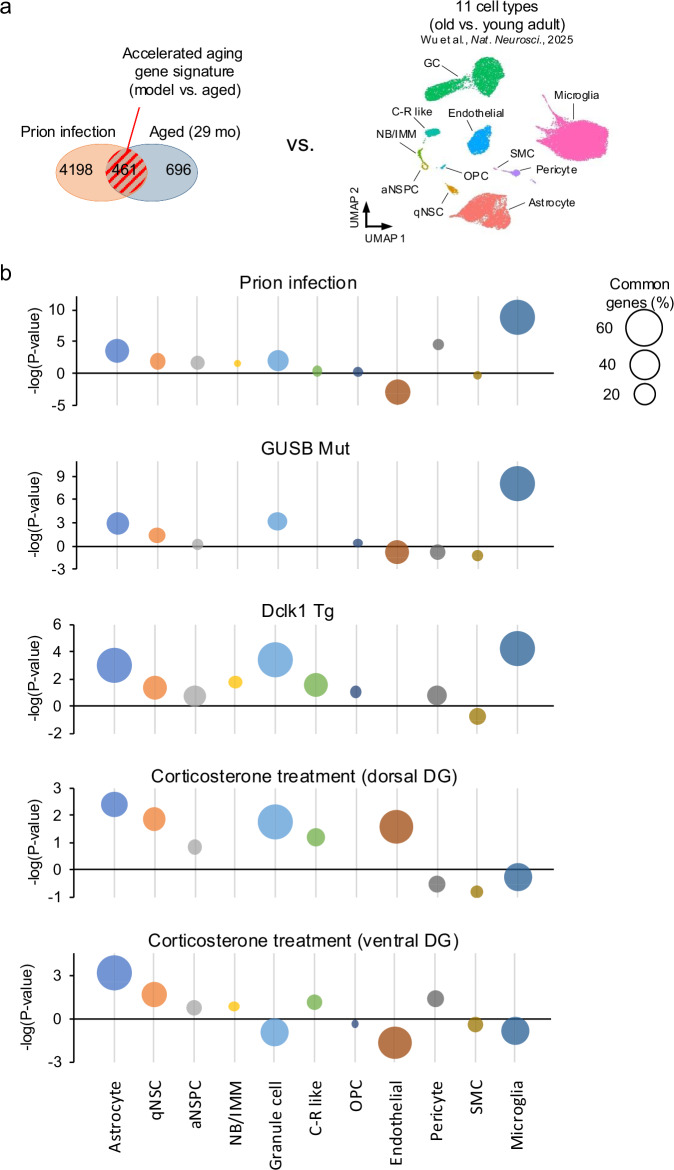
Cell-type contribution analysis of the gene expression signature related to accelerated aging. **a** Expression patterns of shared genes between each mouse model and aged mice (shaded) were compared with aging-related gene signatures from 11 cell types in the DG (16–21-month-old aged mice compared to 3-month-old young adult mice). **b** Plot showing the degree of similarity of gene expression patterns. The y-axis represents –log(overlap P-value), where higher values indicate greater similarity. Bubble size indicates the percentage of common genes relative to the accelerated aging signature genes (shaded in **a**).

Finally, we estimated which cell types contribute to accelerated aging-related gene expression signatures. A previous study conducted single-cell RNA-sequencing analysis of aging in the mouse DG and identified 11 distinct cell types [36]. Utilizing the data resource, we obtained the DEGs in old mice compared to young adult mice for each cell type (Table S26) and then compared them with gene expression changes associated with accelerated ageing in each mouse model (Fig. 5a). In prion-infected, GUSB mutant and Dclk1 Tg mice, a strong contribution from microglia was observed, along with moderate contributions from astrocytes and GCs (Fig. 5b). In corticosterone-treated mice, contributions from astrocytes (dorsal and ventral DG) and GCs (dorsal DG) were also detected, whereas microglial contribution appeared to be lower.

### Transcriptomic hyper-maturity in the hippocampus of patients with neuropsychiatric disorders

Finally, we investigated whether the hyper-maturity phenotype might also be observed in the hippocampus of patients with psychiatric disorders frequently comorbid with anxiety symptoms, including SZ, BD, and MDD [43–45]. Transcriptomic data for these disorders were obtained from three studies [37–39] and compared with DEGs associated with postnatal development (20–39 years old vs. 0–5 months old) and aging (over 70 years old vs. 20–39 years old) [40]. The results revealed that the degree and direction of similarity to developmental and aging-related gene expression varied across the disease datasets (Fig. S3). A hyper-maturity phenotype, indicated by a significant positive correlation with postnatal development, was observed in one BD dataset (Fig. S3i) and one MDD dataset (Fig. S3k). An accelerated aging, defined by a significant positive correlation with aging, was identified in two SZ datasets (Fig. S3b, h), one BD dataset (Fig. S3j), and one MDD dataset (Fig. S3l). In contrast, two SZ datasets (Fig. S3a, m) and two BD datasets (Fig. S3c, o) showed significant negative correlations with postnatal development, suggesting a transcriptomic pseudo-immaturity phenotype. One MDD dataset demonstrated a negative correlation with aging (Fig. S3r). While certain datasets demonstrated characteristics of hyper-maturity or accelerated aging, consistent findings regarding maturation abnormalities were not observed across diseases or studies. Such variability may, in part, be attributable to confounding factors such as medication effects.

## Discussion

This study provided transcriptomic evidence that various genetic and environmental manipulations can induce hyper-maturity and accelerated aging in the mouse hippocampus. The contrasting manifestations of anxiety-related behaviors in the hippocampal hyper-maturity and immaturity mouse models suggest that maturational plasticity in the hippocampus may be a potential correlate of this type of behavior.

It should be noted that the hippocampal subregions examined were not completely matched; the identified datasets included those from the DG and the entire hippocampus (Table S5). Despite the limitation, this study provides evidence that hyper-maturation of the hippocampus could be induced by multiple genetic and environmental factors.

It is well established that new neurons are generated in the DG of the hippocampus in rodents and non-human primates during adulthood, a phenomenon known as adult hippocampal neurogenesis (AHN). Consequently, one might hypothesize that the detection of a hyper-maturity phenotype in transcriptomic data from bulk hippocampal tissues could be attributed to a reduction in AHN. Indeed, studies suggest that corticosterone-treated mice exhibit decreased AHN, as indicated by BrdU incorporation analysis [46, 47]. However, in other hyper-maturity models, both Sert heterozygous KO mice and homozygous KO mice show no changes in AHN [48], while SAMP8 mice exhibit increased AHN during adulthood [49]. Regarding AHN in iDG mouse models, Camk2a KO mice [1], Cn KO mice [12], and pilocarpine-treated mice [50] display increased AHN in similar analyses, whereas Snap25 Tg mice exhibit decreased AHN [8]. These observations suggest that neither decreased nor increased AHN necessarily correlates with the hippocampal hyper-maturity or immaturity phenotype. Therefore, upregulation or downregulation of AHN may not be a major contributing factor to transcriptomic immaturity or hyper-maturity in the hippocampus. This may be attributed to the relatively small number of neurons generated through AHN in adulthood; proliferating cells are estimated to account for up to 0.5% of the total GC population in a few-month-old mammalian animals [51, 52]. Instead, alterations in pre-existing mature GCs, which constitute the majority of cells in the adult DG, may underlie the observed transcriptomic immaturity in the bulk hippocampal tissues. Single-cell transcriptomics may provide further insights into whether stem/progenitor cell populations are altered in these mouse models.

Regarding the functional implications of hippocampal hyper-maturity, we found a potential link to increased anxiety-like behaviors, while its association with other behavioral domains remains unclear, representing a limitation of the present study. Observations of decreased anxiety-like behaviors in hippocampal immaturity models further support this idea (Table S24). However, this study is correlational in nature and does not establish a causal relationship between hippocampal maturation abnormalities and changes in anxiety-related behaviors. Moreover, it should be noted that immaturity models do not necessarily exhibit decreased anxiety-like behavior. For example, FLX-treated mice [53] and Snap25 KI mice [8] have been reported to display increased anxiety-like behavior in the open field and light-dark transition tests. Therefore, it is possible that hippocampal maturation abnormality is associated with anxiety-related behavior, that it is unrelated, or that it contributes in combination with other brain abnormalities. As the amygdala is considered a central region involved in regulating anxiety [54, 55], it would be of interest to investigate whether molecular and functional alterations are present in the amygdala of these mouse models with hippocampal maturation abnormalities. If future research establishes that hippocampal hyper-maturity is causally involved in the manifestation of anxiety-like behaviors, this phenotype might represent a potential target for interventions aimed at mitigating the onset or progression of neuropsychiatric disorders involving anxiety.

The present study did not identify mechanisms that are commonly involved in the hippocampal hyper-maturity phenotype across diverse mouse models. We specifically tested the possible roles of neural excitation/suppression and corticosterone-related stress exposure, but none of these factors robustly characterized the hyper-maturity models. Nevertheless, our mouse experiments clearly demonstrated that chronic elevation of corticosterone is one causal factor driving hippocampal hyper-maturity. We also showed that at least a subset of patient populations exhibits transcriptomic features indicative of hippocampal hyper-maturity, and several studies have indeed reported elevated systemic glucocorticoid (primarily cortisol) levels in patients with SZ, MD, or anxiety disorder [56–60]. Future studies assessing the relationship between glucocorticoid levels and maturity levels on an individual basis may help to clarify this link more definitively.

SAMP8 mice are generally used as a model for studying brain aging, as they exhibit age-related changes early in life, such as increased oxidative stress and gliosis [61]. Unexpectedly, however, our analysis indicated that, in terms of hippocampal transcriptomic changes, SAMP8 mice were more closely associated with the overrepresentation of postnatal development than with accelerated aging. Our findings may offer a novel perspective on the use of this mouse model in brain aging research, which may not be apparent when focusing solely on individual molecules, particularly specific aging-related markers.

We found that GUSB mutant mice were preferentially associated with accelerated aging. Mutations in the gene encoding the lysosomal enzyme GUSB are known to cause mucopolysaccharidosis type VII, a lysosomal storage disorder that leads to the accumulation of mucopolysaccharides in tissues and results in various abnormalities, including skeletal dysplasia and intellectual disability, beginning in the fetal and early childhood stages [62]. Lysosomal is also implicated in aging-related neurodegenerative disorders, and even in normal aging processes [63, 64]. In this context, our findings align with previous observations, suggesting that GUSB mutant mice may exhibit aging-like cellular and organelle-level phenotypes due to exacerbated lysosomal impairment. Corticosterone-treated mice present another model of hippocampal accelerated aging. In our previous studies, a comprehensive behavioral test battery demonstrated that this model exhibits not only increased anxiety-like behavior but also a range of aging-like behavioral alterations, such as reduced motor function, decreased locomotor activity in a novel environment, decreased acoustic startle response activity, and impaired social interaction [29, 65, 66]. Together, GUSB mutant and corticosterone-treated mice recapitulate key molecular and behavioral features of aging and may therefore serve as useful models for studying accelerated aging.

In conclusion, hippocampal maturation cannot only be arrested at or reversed to an immature state but also excessively advanced due to various genetic and environmental insults. If such abnormalities in hippocampal maturation contribute to changes in anxiety-related behaviors, elucidating their underlying mechanisms may enhance our understanding of the molecular basis of neuropsychiatric disorders and the aging process involving anxiety, and aid in the development of novel therapeutic and anti-aging strategies.

## Supplementary information

Supplementary Figures 1–3

Supplementary Tables 1–26

## Data Availability

The gene expression data analyzed in this study are included in Supplementary Tables.
