# Supplementary material for: Hyper-maturity and accelerated aging in the hippocampus of mouse models of neuropsychiatric disorders with anxiety-like behavior
Source: Neuropsychopharmacology. 2025 Oct 27;51(5):856–67. doi: 10.1038/s41386-025-02237-6 (PMC13013628; doi:10.1038/s41386-025-02237-6)
Supplement: Supplementary file 1 — Supplementary Figures 1–3 [file 41386_2025_2237_MOESM1_ESM.pdf]

Supplementary Figures

Hyper-maturity and accelerated aging in the hippocampus of mouse models of neuropsychiatric disorders with anxiety-like behavior

Hideo Hagihara, Ph.D.[1*]; Hisatsugu Koshimizu, Ph.D.[1,2]; Satoko Hattori, Ph.D.[1,3]; Hirotaka Shoji, Ph.D.[1]; Miho Tanaka, Ph.D.[4]; Kazutaka Ikeda, Ph.D.[4,5]; Tsuyoshi Miyakawa, Ph.D.[1*]

1. Division of Systems Medical Science, Center for Medical Science, Fujita Health University, Toyoake, Aichi, Japan
2. Office of Research Administration, Fujita Health University, Toyoake, Aichi, Japan
3. Research Creation Support Center, Aichi Medical University, Nagakute, Aichi, Japan
4. Addictive Substance Project, Tokyo Metropolitan Institute of Medical Science, Setagaya-ku, Tokyo, Japan
5. Department of Neuropsychopharmacology, National Institute of Mental Health, National Center of Neurology and Psychiatry, Kodaira, Tokyo, Japan

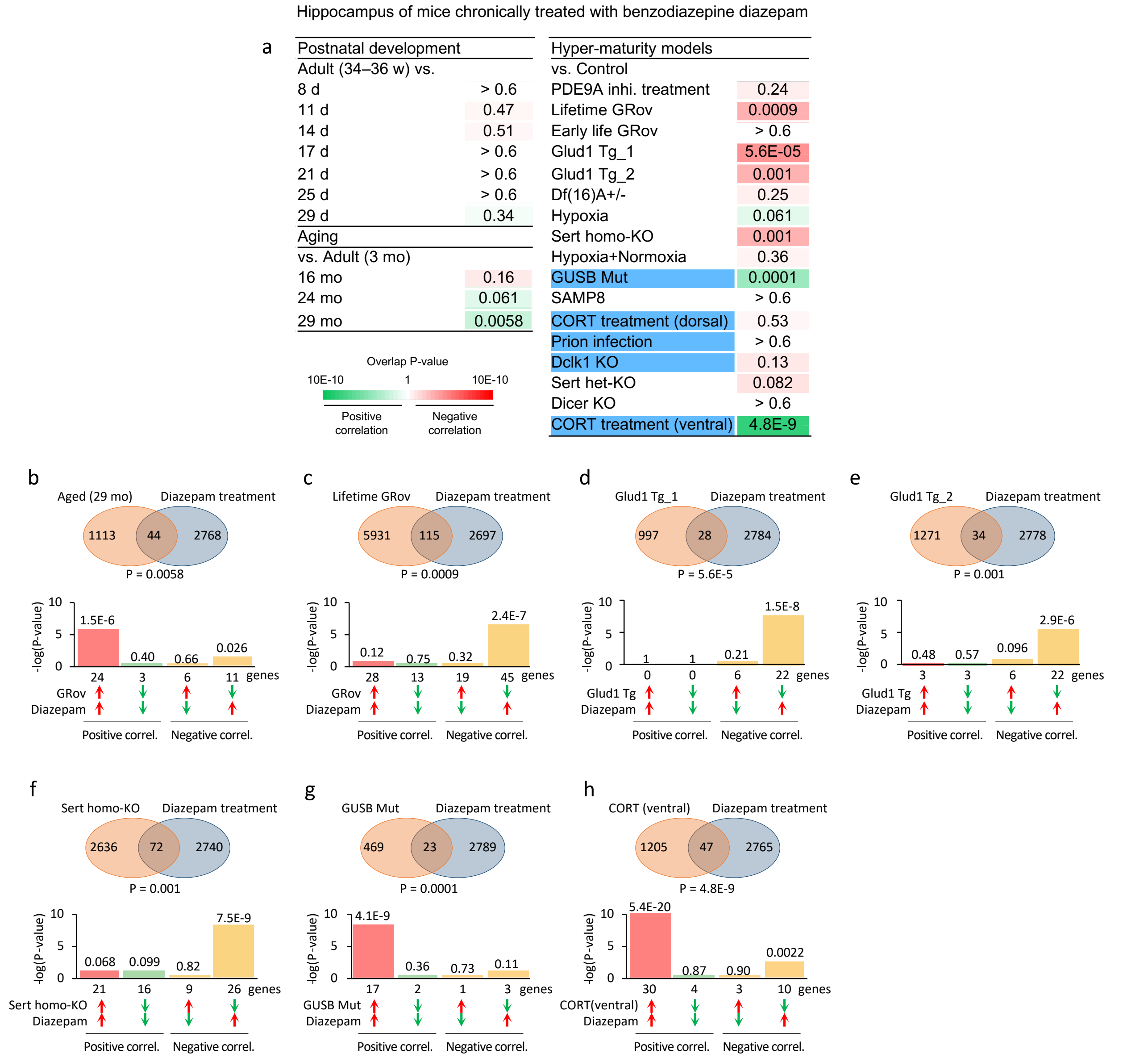

Figure S1. Comparisons of gene expression patterns between mice treated with anxiolytic benzodiazepine diazepam and infant, aged, and hyper-maturity model mice. a. Overlap P-values calculated using the Running Fisher test to compare gene expression in diazepam-treated mice with those from postnatal development (Adult vs. 8–29 days), aging (16–29 months vs. Adult), and hyper-maturity model mice (model vs. control). The color scale indicates the direction of correlation: red for negative and green for positive. b–h. Venn diagrams and bar graphs for dataset pairs in panel (a) that exhibited statistical significance (overlap P-value < 0.05).

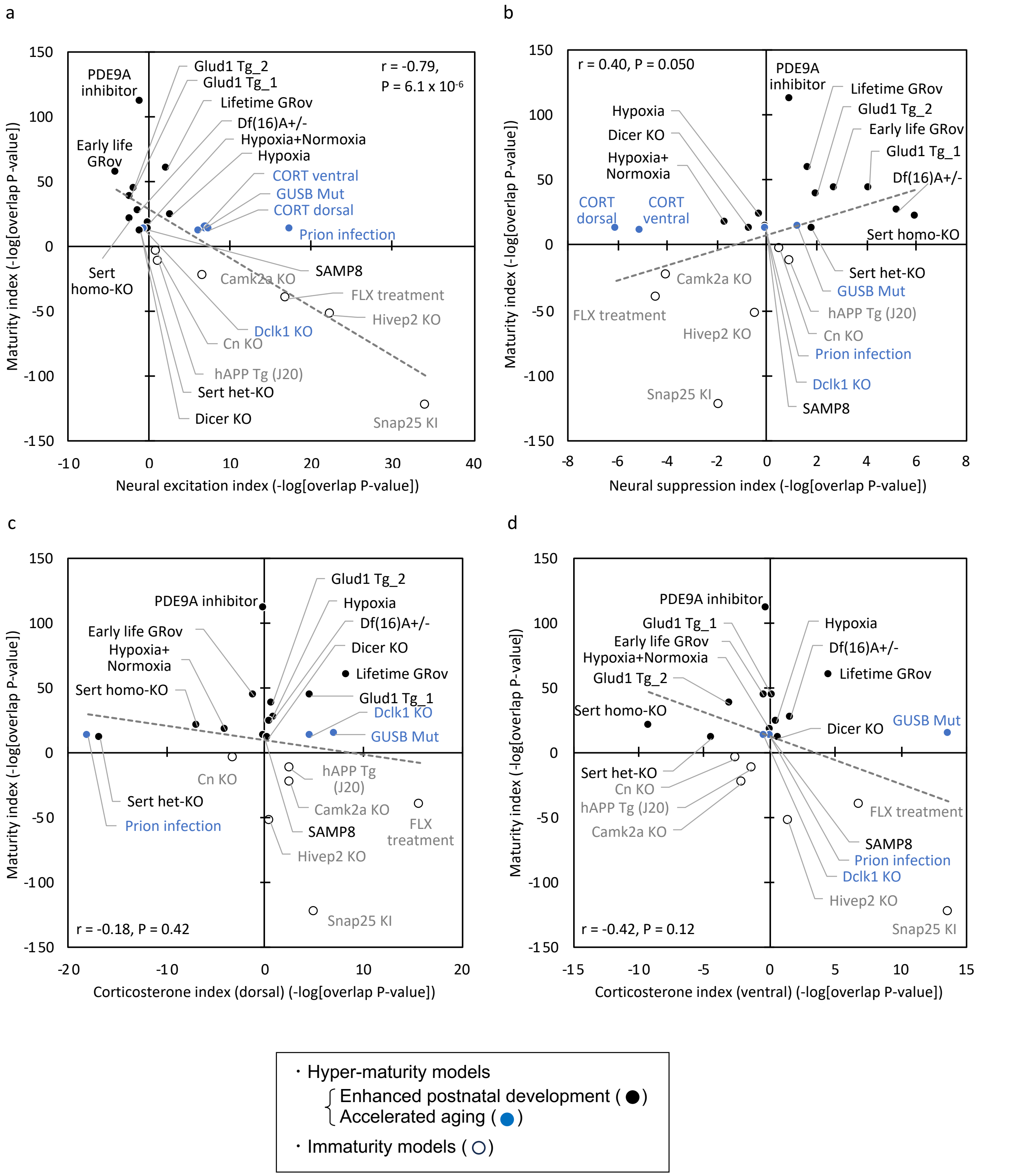

Figure S2. Relationships of neural excitation, neural suppression and corticosterone-related stress with hippocampal hyper-maturity. Scatter plots showing correlations between the maturity index and the neural excitation index (a), neural suppression index (b), and corticosterone index (dorsal DG, c; ventral DG, d).

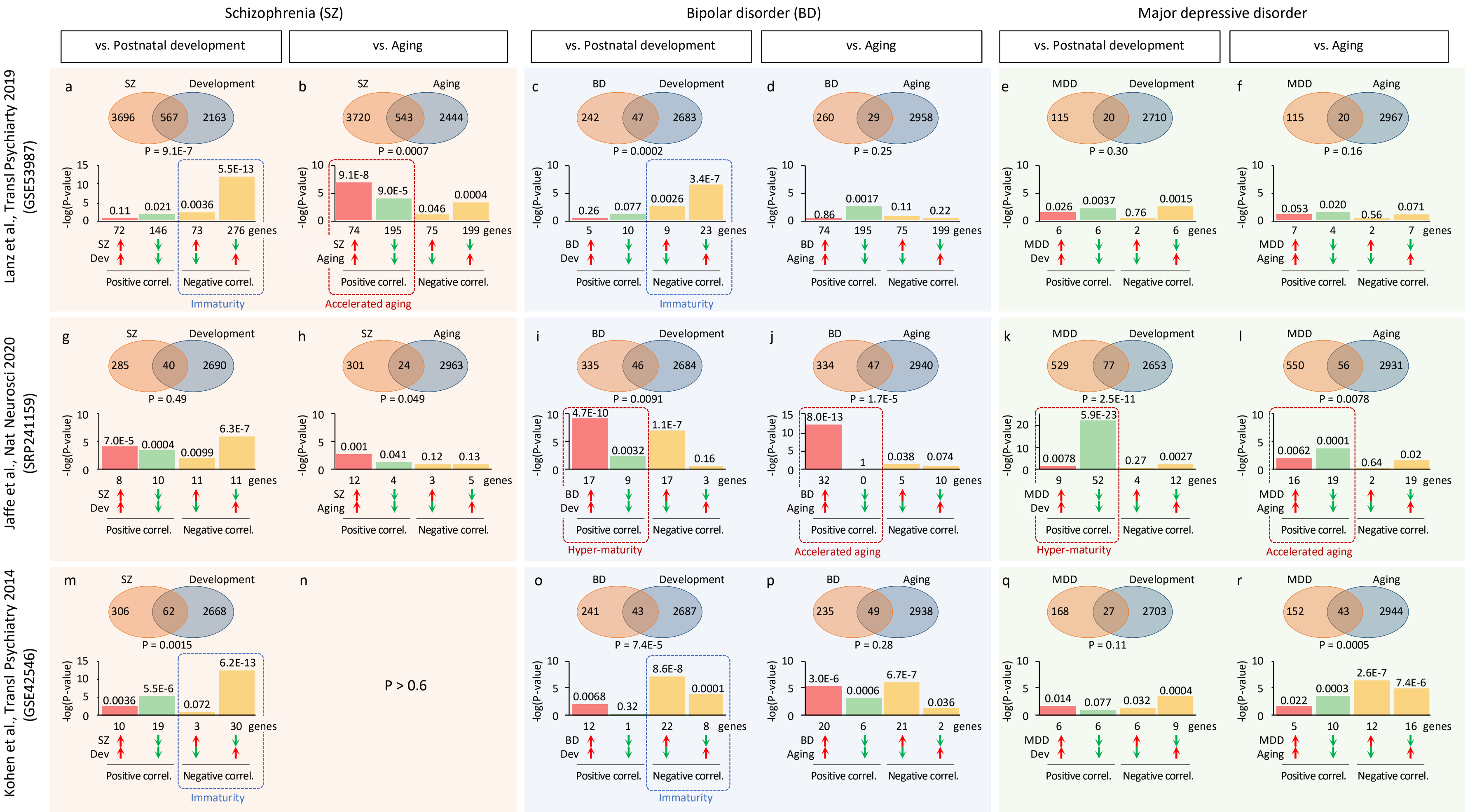

Figure S3. Transcriptomic hyper-maturity in the hippocampus of patients with neuropsychiatric disorders. a–r. Venn diagrams and bar graphs showing comparisons between disease datasets (patients vs. controls) and datasets of postnatal development (20–39 years old vs. 0–5 months old) or aging (over 70 years old vs. 20–39 years old). Transcriptome data of schizophrenia (SZ), bipolar disorder (BD), and major depressive disorder (MDD) were obtained from three studies and analyzed. In the comparison shown in panel (**n**), the overlap P-value was greater than 0.6, and therefore a Venn diagram and bar graph were not generated in the BaseSpace platform.
